# sFgl2 gene-modified MSCs regulate the differentiation of CD4^+^ T cells in the treatment of autoimmune hepatitis

**DOI:** 10.1186/s13287-023-03550-x

**Published:** 2023-11-03

**Authors:** Wenbin Ji, Weiwei Wang, Peiyuan Li, Yanhong Liu, Baotong Zhang, Feng Qi

**Affiliations:** 1 Department of General Surgery, Tianjin Medical University General Hospital, No. 154, Anshan Road, Heping District, Tianjin, 300052 China; 2 Department of General Surgery, Tianjin Medical University Baodi Clinical College, Guangchuan Road, Baodi, Tianjin, 301800 China; 3 Department of General Surgery, Tianjin Union Medical Center, Tianjin, China; 4 Tianjin Key Laboratory of General Surgery in Construction, Tianjin Union Medical Center, Tianjin, 300121 China

**Keywords:** Autoimmune hepatitis, Mesenchymal stem cell, Soluble fibrinogen-like protein 2, Fcγ receptor IIB, CD4^+^ T cell

## Abstract

**Background:**

Autoimmune hepatitis (AIH) is a T-cell-mediated autoimmune liver disease that can lead to liver injury and has a poor long-term prognosis. Mesenchymal stromal cells (MSCs) have immunosuppressive effects and can treat AIH. CD4^+^ T cells express the unique inhibitory Fcγ receptor (FcγRIIB), which is the only receptor for the immunosuppressive factor soluble fibrinogen-like protein 2 (sFgl2). This study aimed to examine the therapeutic effect of sFgl2 gene-modified MSCs (sFgl2-MSCs) on AIH.

**Methods:**

MSCs were obtained from the inguinal fat of mice and cocultured with CD4^+^ T cells sorted from mouse spleens. FcγRIIB expression on CD4^+^ T cells was determined by flow cytometry. sFgl2 expression in MSCs transfected with lentiviral vectors carrying the Fgl2 gene and a green fluorescent protein-encoding sequence was determined by enzyme-linked immunosorbent assay. The percentages of Th1 cells Th17 cells and regulatory T cells (Tregs) were determined by flow cytometry And the levels of p-SHP2 and p-SMAD2/3 were detected by Western blotting after the cells were cocultured with MSCs for 72 h. After locating MSCs by in vivo imaging Con A-induced experimental AIH mice were randomly divided into 4 groups and administered different treatments. After 24 h histopathological scores liver function and cytokine levels were examined and the proportions of CD4^+^ T cells CD8^+^ T cells Tregs Th17 cells and Th1 cells in the spleen and liver were determined by flow cytometry. In addition immunohistochemical staining was used to detect the liver infiltration of T-bet-, Foxp3- and RORγ-positive cells.

**Results:**

FcγRIIB expression on CD4^+^ T cells was upregulated after coculture with MSCs. After coculture with sFgl2-MSCs, the proportion of Tregs among CD4^+^ T cells increased, the proportion of Th17 and Th1 cells decreased, and the levels of p-SHP2 and p-SMAD2/3 increased. In vivo, sFgl2-MSCs significantly improved liver function, decreased liver necrosis area, decreased tumor necrosis factor-α, interleukin (IL)-1β and IL-6 expression, increased IL-10 expression, reduced liver infiltration of CD4^+^ T and CD8^+^ T cells, increased the proportion of Tregs and reduced the proportions of Th17 and Th1 cells in mice.

**Conclusion:**

By promoting Tregs differentiation and inhibiting Th17 and Th1 cell differentiation, sFgl2 gene-modified MSCs have a more powerful therapeutic effect on Con A-induced experimental AIH and may represent a strategy for the clinical treatment of AIH.

**Supplementary Information:**

The online version contains supplementary material available at 10.1186/s13287-023-03550-x.

## Introduction

Autoimmune hepatitis (AIH) is one of the most common causes of life-threatening T-cell-mediated liver damage in humans [1]. AIH triggers T-cell-mediated autoimmune responses against liver autoantigens. CD4^+^ and CD8^+^ T cells are the major T lymphocytes that infiltrate the liver [1, 2]. CD4^+^ T cells are considered to be the key cells in the occurrence and development of AIH, and liver damage in AIH may be caused and mediated by CD4 T lymphocytes that recognize self-antigens [3].Presentation of peptides contained by HLA class II molecules by antigen-presenting cells to naive CD4 helper T cells is the initiation of pathogenesis [4–6].

AIH remains a serious health challenge worldwide because it can eventually result in liver cirrhosis, liver cancer, and even death [6–8]. AIH treatment remains a clinical issue that cannot be ignored. Therefore, finding a potential therapeutic strategy is urgent for the treatment of acute hepatitis.

Mesenchymal stromal cells (MSCs) have the potential to self-renew, differentiate into multiple cell lineages and modulate immune properties [9, 10]. MSCs come from a wide range of sources, repair tissue damage and are thought to have great potential in cell therapy [11–13]. MSCs can accumulate at sites of inflammation and play immunomodulatory roles [14]. In liver disease, MSCs play multiple roles in liver regeneration by inhibiting hepatocyte apoptosis, reversing liver fibrosis and reducing inflammation [15], and several studies have used MSCs to treat AIH [16, 17]. However, the curative effect of using MSCs alone still needs to be improved, and it is necessary to seek ways to enhance the immune regulatory function of MSCs [18–20].

Fcγ receptors play a key role in controlling innate and humoral immunity by activating the effector function of antibodies. Fcγ receptor II b (FcγRIIb, CD32b) is a low-affinity Fcγ receptor and is the only inhibitory Fcγ receptor expressed on a variety of immune cells, including B cells, macrophages, dendritic cells (DCs) and granulocytes [21].

Recent studies have shown that CD32b is also present and functional on T cells, and soluble fibrinogen-like protein 2 (sFgl2) is a key functional ligand of CD32b [22]. sFgl2 is a key immunosuppressive factor secreted by regulatory T cells (Tregs) and an important effector molecule of Tregs [23]. sFgl2 has a significant immunosuppressive effect and has been widely used in the study of organ transplantation, inflammatory bowel disease, sepsis, tumors, and parasitic diseases [24, 25].

CD32b is the only receptor of sFgl2 [26], and this study revealed that CD32b expression on CD4^+^ T cells could be induced by coculture with MSCs. Therefore, we aimed to examine the potential of sFgl2 gene-modified MSCs in the treatment of experimental AIH, which may provide a new strategy for the clinical treatment of AIH.

## Methods

### Animals

Healthy adult male C57BL/6 J mice weighing 20–21 g were used in this study. The animals were housed in wire-bottomed, wire-lidded cages, allowed access to chow and water ad libitum and were acclimatized for 1 week before experiments in a temperature-controlled room (25 ± 2 °C) with a 12-h light/dark cycle. All animal experiments were performed according to the Chinese Council on Animal Care guidelines and the basis of protocols approved by the Animal Ethical and Welfare Committee of Tianjin Medical University General Hospital (Tianjin, China) (No. IRB2021-DW-41).

### Isolation of MSCs

Fat was isolated from the groins of 4-week-old C57BL/6 J mice on a clean bench and transferred to 15 mL centrifuge tubes (Corning, New York, USA) containing 5 mL of DMEM/F12 medium (Gibco, MA, USA). Collagenase type I (1 mg/mL) (Solarbio Life Science, Beijing, China) was added and incubated at 37 °C for 1.5 h under shock conditions (200 rpm). Fully digested adipose tissue was filtered through a 40-μm mesh strainer and centrifuged at 1500 rpm for 5 min to obtain cells, and this process was repeated once. The cells were resuspended in DMEM/F12 medium (Corning, New York, USA) containing 1% antibiotic cocktail (Solarbio Life Sciences, Beijing, China) and 15% fetal bovine serum, seeded on 6 cm dishes (Corning, New York, USA) and incubated in a 37 °C incubator with 5% CO_2_. The medium was changed every 48 h. The isolated MSCs were used in subsequent experiments.

### Transfection of sFgl2 into MSCs

Second-generation MSCs were seeded in 6-well plates at a density of 3.0 × 10^5^/well and cultured until the fusion rate reached 60%. Then, a 1-mL transfection system composed of 40 μL of enhanced infection solution, 10 μL of lentiviral vectors carrying the Fgl2 gene and encoding green fluorescent protein (Ubi-MCS-3FLAG-CBh-gcGFP-IRES-puromycin, 5.0 × 10^8^ TU/mL) (Genchem, Shanghai, China), and 950 μL of DMEM/F12 medium containing 15% fetal bovine serum was prepared for viral transfection. After 12 h at 37 °C, the transfection medium was replaced with complete medium. Three days later, the stably transfected MSCs (sFgl2-MSCs) were screened with 2 μg/mL puromycin (Solarbio Life Science, Beijing, China). The cells and cell supernatants were used for subsequent experiments.

### Coculture of CD4^+^ T cells with MSCs and design of experimental groups in vitro

Naïve CD4^+^ T cells were isolated from C57BL/6 mouse splenocytes by a CD4^+^ microbead kit (Miltenyi, Germany). CD4^+^ T cells (3.0 × 10^5^) were plated on 96-well plates that were precoated with CD3 (5 µg/mL) (eBioscience Inc. USA) and incubated for 2 h in a 100 µL system, and CD28 (1 µg/mL) (eBioscience Inc. USA) and IL2 (15 ng/mL) (eBioscience Inc. USA) were added. CD4^+^ T cells were incubated at 37 °C in CO_2_ for 12 h to obtain activated CD4^+^ T cells [27, 28]. Transfected or untransfected MSCs were collected and resuspended. Then, we cocultured activated CD4^+^ T cells (3.0 × 10^5^) with MSCs (3.0 × 10^4^) in a 48-well plate. After 72 h [29, 30], CD4^+^ T cells were collected in different tubes for flow cytometric analysis to detect the proportions of CD4^+^ T-cell subsets. The experiment included 6 groups: the untreated group, wild-type MSC (WT-MSC) group, negative control MSC (NC-MSC) group, sFgl2-MSC group, sFgl2-MSC + anti-CD32b group and recombinant Fgl2 (rFgl2) group.

The control group contained only stimulated CD4^+^ T cells, the rFgl2 group was supplemented with rFgl2 (600 ng/mL, R&D Systems, USA) plus stimulated CD4^+^ T cells, and the anti-CD32b group was supplemented with anti-CD32b antibody (15 µg/mL, BioXcell, USA) plus sFgl2-MSCs. The concentrations of rFgl2 and anti-CD32b antibodies were obtained from preliminary experiments.

### Con A-induced model and experimental groups in vivo

Caudal intravenous injection of Con A has been used to induce experimental AIH to generate models [31–33]. Liver damage was induced by injecting Con A (Solarbio Science and Technology Co, Ltd, China) via the tail vein at a dose of 15 mg/kg [34, 35] dissolved in 150 µL phosphate-buffered saline (PBS). Thirty minutes after the administration of Con A, 30 mice were randomly assigned to the following five groups (n = 6): (i) healthy mice injected with 150 µL PBS (without Con A) via the tail vein; (ii) PBS-treated mice injected with 150 µL PBS via the tail vein; (iii) WT-MSC-treated mice intravenously injected with MSCs (1 × 10^6^ cells/mouse) suspended in 150 µL of PBS; (iv) NC-MSC-treated mice intravenously injected with MSCs (1 × 10^6^ cells/mouse) suspended in 150 µL PBS; and (v) sFgl2-MSC-treated mice intravenously injected with sFgl2-MSCs (1 × 10^6^ cells/mouse) in the same manner. All mice were euthanized 24 h after the administration of Con A. Animals were euthanized by carbon dioxide (CO_2_) inhalation. CO_2_ (100%) was delivered into an enclosed space at a replacement rate of 40% per minute until the mice lost their spontaneous respiration ability and died. Blood, livers, and spleens were collected and stored for further analysis.

### Flow cytometry

Antibody staining and flow cytometry were performed as described previously [36]. Third-generation MSCs were collected and stained with anti-CD29 (fluorescein isothiocyanate, FITC), anti-CD44 (phycoerythrin, PE), anti-CD45 (PE), anti-CD34 (allophycocyanin, APC), and anti-SCA-1 (APC).

CD4^+^ T cells were collected from in vitro cocultures, resuspended and stained with fluorescent antibodies, including anti-CD4 (FITC), anti-CD25 (PE), anti-Foxp3 (APC), anti-IL-17A (PerCP-Cyanine 5.5), anti-interferon (IFN-γ, PE), and anti-CD32b (PE, eBioscience, USA).

The spleens were collected, prepared into single-cell suspensions and used for staining. The mouse liver was flushed with 0.9% normal saline via the inferior vena cava to remove residual blood and prevent blood lymphocytes from interfering with the results of subsequent experiments. After the mouse liver was ground, a single-cell suspension was obtained through a 200-mesh filter. Hepatic lymphocytes were isolated by Percoll gradient separation (top Percoll layer: 40%; bottom Percoll layer: 70%) and divided into different test tubes for staining [37]. The fluorescent antibodies used included anti-CD3 (FITC), anti-CD4 (APC), anti-CD8 (PerCP-Cyanine 5.5), anti-CD4 (FITC), anti-CD25 (PE), anti-IL-17A (PerCP/Cyanine 5.5), and anti-Foxp3 (APC). All cell staining procedures were based on the manufacturer’s instructions.

To exclude nonspecific staining caused by dead cells, a distinction needed to be made between living cells and dead cells. Viable cells were identified with a zombie dye (APC-Cyanine 7). All antibodies for flow cytometry were purchased from BioLegend (USA) unless otherwise stated. The proportions of various phenotypes of immune cells were analyzed using FlowJo software.

### MSC tracking in vivo

To determine whether MSCs could reach the site of inflammation, we used live organ imaging. MSCs were stained in vitro using CM-Dil (Ex/Em = 553/570 nm, Thermo Fisher, USA) according to the manufacturer’s instructions. Then MSCs were transplanted into mice through the tail vein 30 min after the injection of Con A. After 12 h, the liver and lung were harvested and collected fluorescence images.

### Measurement of alanine aminotransferase (ALT) and aspartate aminotransferase (AST)

The serum activities of ALT and AST were used to evaluate liver function and liver damage and were measured using ALT and AST assay kits (Nanjing Jiancheng, Nanjing, China) according to the manufacturer’s instructions.

### H&E staining

The liver was flushed with 0.9% normal saline in the inferior vena cava to prevent blood residue and then harvested. The liver tissues were fixed with 10% formalin, embedded in paraffin and cut into 5 μm sections for H&E staining. Then, the degree of liver injury was blindly evaluated by two pathologists based on the liver Knodell scoring system, and the area of necrosis in liver tissue was measured using iViewer.

### Western blotting

Total protein was extracted from CD4^+^ T cells in the coculture system using RIPA lysis buffer (Solarbio, Beijing, China) containing protease inhibitors (APExBIO Technology LLC, USA). The protein samples were diluted to the same final total protein concentration and then subjected to 10% SDS‒PAGE; the proteins were transferred to polyvinylidene fluoride membranes (Merck Millipore, USA). Monoclonal antibodies for detecting SHP2 (1:1000) (Affinity, China), phospho-SHP2 (1:1000) (Affinity, China), SMAD2/3 (1:1000) (Cell Signaling Technology, USA), phospho-SMAD2/3 (1:1000) (Cell Signaling Technology, USA), GAPDH (1:1000) (Affinity, China) and β-actin (1:1000) (Affinity, China) were added to the membranes and incubated at 4 °C overnight. The membranes were washed and incubated with the corresponding diluted secondary horseradish peroxidase (HRP)-marked antibodies (1:2000) (Affinity, China) at room temperature for 1 h. Finally, the blots were developed with ECL solution and photographed with an exposure instrument.

### Immunohistochemical staining

T-bet, RORγ and Foxp3 in liver tissues were examined with a streptavidin–biotin peroxidase complex-based immunohistochemical technique. The liver tissues were fixed with 10% formalin, embedded in paraffin and cut into 5-μm sections for staining. The liver sections were incubated with the following primary antibodies: anti-T-bet (1:1000, Affinity, China), anti-Foxp3 (1:1000, Affinity, China) and anti-RORγ (1:1000, Affinity, China). Under a light microscope, the numbers of T-bet-, RORγ-, and Foxp3-positive cells in 5 different fields (400 ×) in each section were counted.

### Enzyme-linked immunosorbent assay (ELISA)

To determine whether sFgl2 could be secreted by sFgl2-MSCs, we measured sFgl2 in the supernatant of sFgl2-MSCs using an ELISA kit (Uscn Life Science Inc., Wuhan, China).

To determine the levels of inflammatory cytokines in each group, blood samples were collected and centrifuged at 4 °C and 1000 × g for 20 min. The levels of interleukin 10 (IL-10), interleukin 6 (IL-6), interleukin 1β (IL-1β), and tumor necrosis factor-α (TNF-α) in the serum were measured using an ELISA kit (ABclonal, Wuhan, China) according to the manufacturer’s protocol.

### Statistical analysis

The data are expressed as the mean ± standard deviation (mean ± SEM). Statistical analyses were performed with SPSS 21.0 (IBM SPSS, Chicago, USA). One-way analysis of variance (ANOVA) was used for group comparisons. The results were considered significant when *P *< 0.05.

## Results

### MSCs induce the expression of CD32b on CD4^+^ T cells

**Fig. 1:**
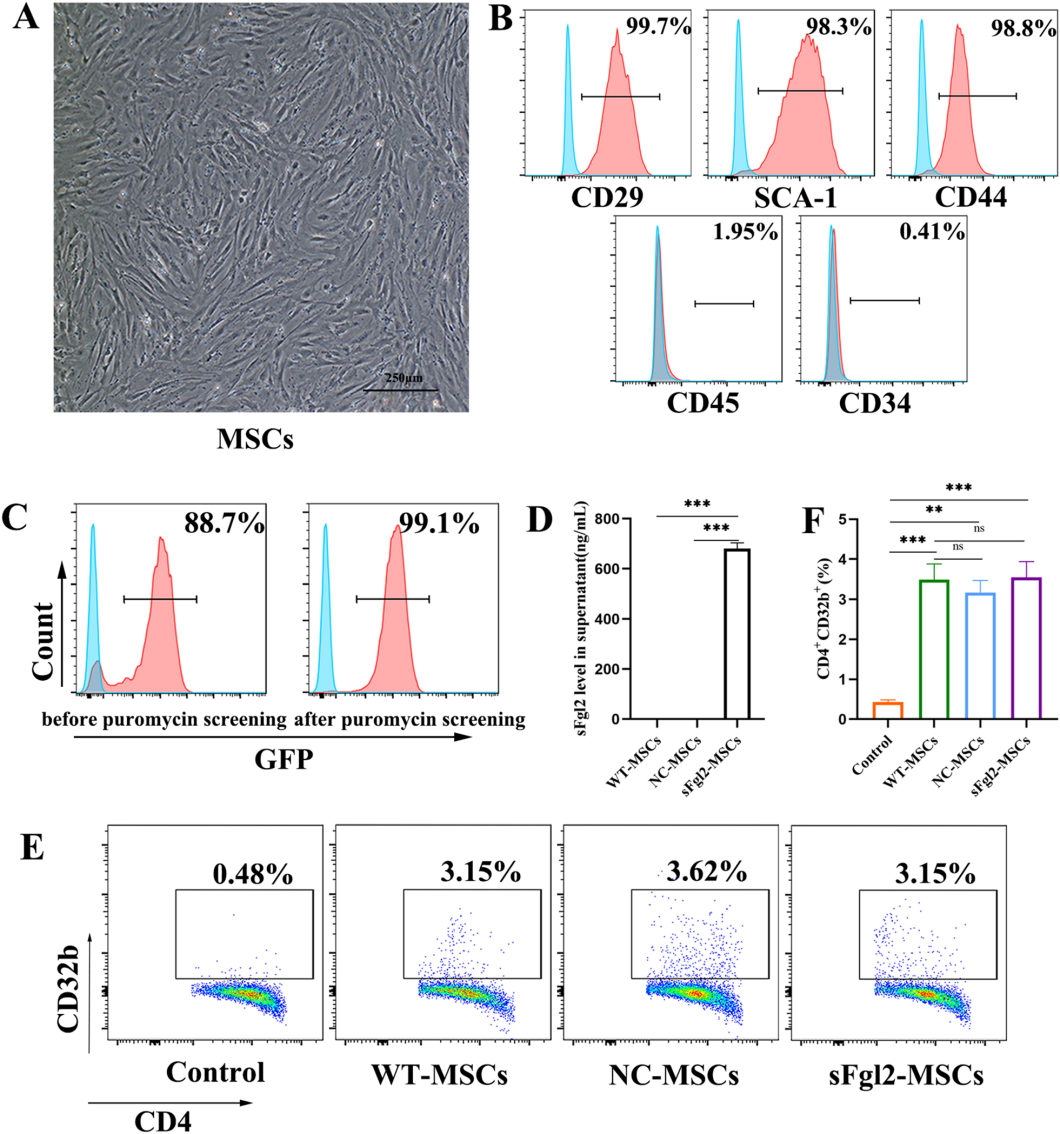
MSCs induced the expression of CD32b on CD4^+^ T cells. **A** Images showing the morphology of mouse-derived MSCs (magnification 40 ×). **B** Flow cytometric analysis of the MSC surface markers CD29, CD34, CD44, CD45, and SCA-1. **C** Flow cytometric analysis of the GFP-positive rates of MSCs before and after puromycin screening. **D** ELISA of sFgl2 concentrations (ng/mL) in the supernatants of different kinds of MSCs. **E** Flow cytometric analysis of CD32b expression levels on CD4^+^ T cells cocultured with modified or unmodified MSCs, n = 3. **F** Proportions of CD32b-positive CD4^+^ T cells in each group. The differences between groups were calculated using ANOVA. ***: *P *< 0.001. Abbreviations: MSCs, mesenchymal stem cells; sFgl2, soluble fibronectin-like protein 2; WT-MSCs, wild-type MSCs; NC-MSCs, negative control MSCs, GFP, green fluorescent protein

Adipose MSCs were obtained from C57BL/6 J mice and exhibited fibroblast-like morphology under a cystoscope (Fig. 1A). Flow cytometry showed that the cells highly expressed CD29, SCA-1 and CD44 but expressed low levels of CD45 and CD34 and could be identified as MSCs (Fig. 1B). After coculture with CD4^+^ T cells, the results showed that CD32b expression on CD4^+^ T cells was significantly increased compared with that in the control group (Fig. 1E and F, *P *< 0.001).

CD32b is known to be the only receptor of sFgl2 [26]. Based on the known immunosuppressive function of sFgl2 [23] and MSCs [14], sFgl2 gene-modified MSCs (sFgl2-MSCs) were constructed by lentiviral transfection, and high-purity sFgl2-MSCs were obtained by puromycin screening (Fig. 1C). To determine the expression of sFgl2 in MSCs, ELISA was used to analyze the cell supernatants, and the results showed that sFgl2-MSCs successfully overexpressed sFgl2, while sFgl2 was not expressed in WT-MSCs (Fig. 1D, *P * < 0.001) or NC-MSCs (Fig. 1D, *P * < 0.001). After coculture with sFgl2-MSCs, the results showed that the expression of CD32b on CD4^+^ T cells was also increased (Fig. 1E and F, *P * < 0.001), and there was no significant difference compared with that in the NC-MSCs (Fig. 1E and F, *P *< 0.001) and WT-MSCs (Fig. 1E and F, *P *< 0.001). Therefore, MSCs can induce the expression of CD32b on CD4^+^ T cells, and this outcome is not affected by viral transfection. In addition, we obtained MSCs with high expression of sFgl2, which laid the foundation for the following experiments.

### sFgl2-MSCs increase the proportion of Tregs and decrease the proportion of Th1 and Th17 cells in CD4^+^ T cells by activating SHP2 and SMAD2/3 in vitro

**Fig. 2:**
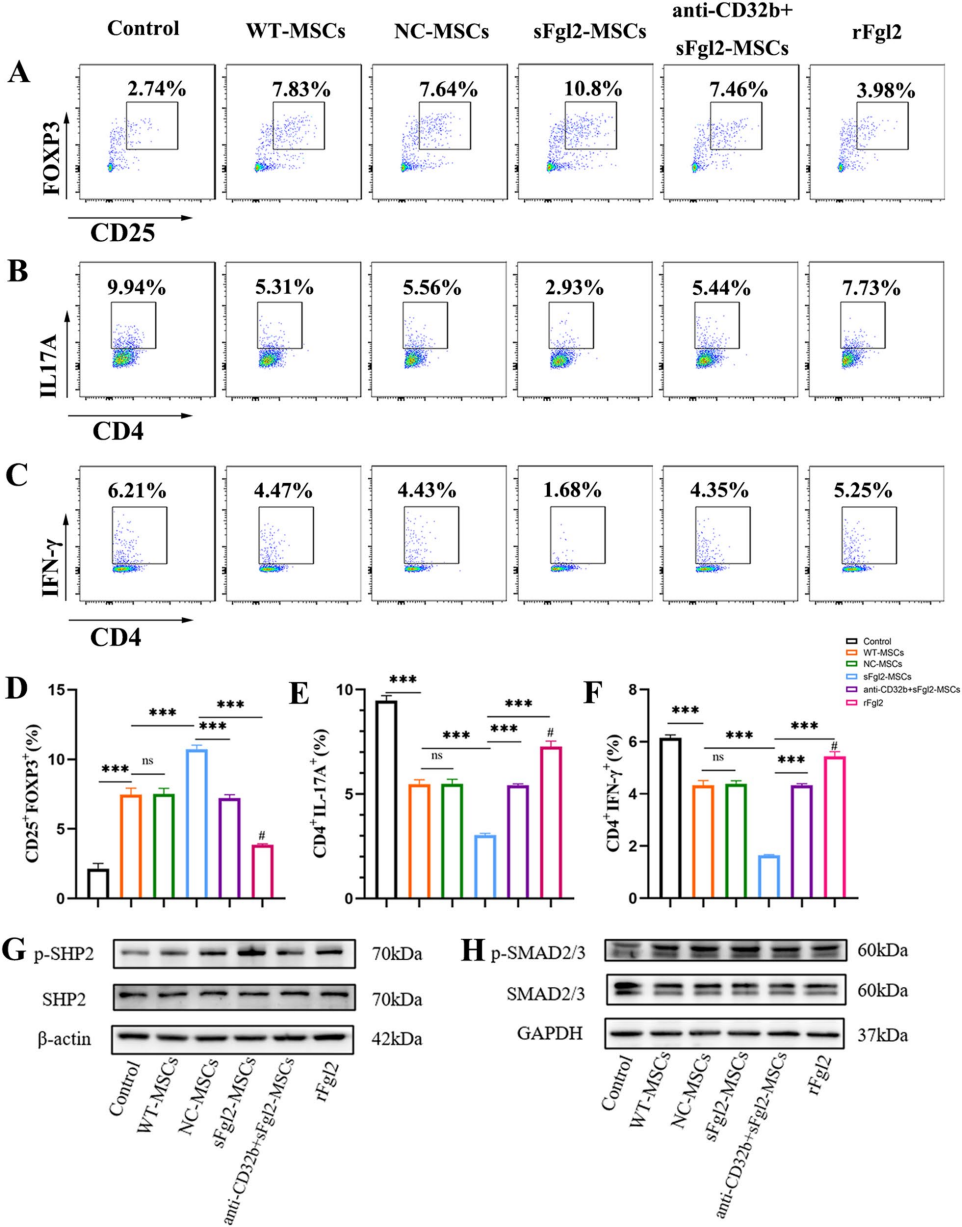
In vitro, sFgl2-MSCs increased the proportion of Tregs and reduced the proportion of Th17 and Th1 cells. **A** Dot plots showing Tregs (CD4^+^CD25^+^Foxp3^+^) among CD4^+^ T cells after coculture for 72 h. **B** Dot plots showing Th17 cells among CD4^+^ T cells after coculture for 72 h. **C** Dot plots showing Th1 cells among CD4^+^ T cells after coculture for 72 h. **D** Proportions of Tregs (CD4^+^CD25^+^Foxp3^+^) in each group, n = 3, **#:** rFgl2 versus control, *P *< 0.05. **E** Proportions of Th17 cells (CD4^+^IL-17A^+^) in each group, n = 3, **#:** rFgl2 versus control, *P *< 0.001. **F** Proportions of Th1 cells (CD4^+^IFN-γ^+^) in each group, n = 3, **#:** rFgl2 versus control, *P *< 0.001. **G** Levels of p-SHP2 in each group. **H** Levels of p-SMAD2/3 in each group. Blots were cropped, and the original blots are presented in Additional file 1: Fig. S1 and Additional file 2: Fig. S2. The differences between groups were calculated using ANOVA. ***: *P *< 0.001. Abbreviations: MSCs, mesenchymal stem cells; sFgl2, soluble fibronectin-like protein 2; WT-MSCs, wild-type MSCs; NC-MSCs, negative control MSCs

After determining that MSCs can increase the expression of CD32b on CD4^+^ T cells, we examined the immunomodulatory function of sFgl2-MSCs by determining the proportion of CD4^+^ T-cell subsets in vitro. After coculture in vitro, the proportion of cells was determined by flow cytometry. The results showed that when only MSCs were present, the proportion of Tregs was higher than that in the control group (Fig. 2A, D, *P * < 0.001), while the proportions of Th17 cells (Fig. 2B and E, *P*  < 0.001) and Th1 cells (Fig. 2C and F, *P*  < 0.001) were lower.

When only rFgl2 was present, the proportion of Tregs was increased compared with that in the control group (Fig. 2A and D, *P*  < 0.05), while the proportions of Th17 cells (Fig. 2B and E, *P * < 0.001) and Th1 cells (Fig. 2C and F, *P *< 0.001) were decreased. Compared with that in the WT-MSC group, the proportion of Tregs in the sFgl2-MSC group containing MSCs and sFgl2 was further increased (Fig. 2A and D, *P* < 0.001), while the proportions of Th17 cells (Fig. 2B and E, *P*  < 0.001) and Th1 cells (Fig. 2C and F, *P* < 0.001) were further decreased. Furthermore, when CD32b was blocked, sFgl2 did not exert its effect, leading to the promotion of Treg differentiation (Fig. 2A and D, *P *< 0.001), and the inhibitory effects of Th17 cells (Fig. 2B and E, *P *< 0.001) and Th1 cells (Fig. 2C and F, *P *< 0.001) were less pronounced than those in the sFgl2-MSC group.

To clarify the possible pathways by which sFgl2-MSCs regulate the differentiation of CD4^+^ T-cell subsets, we first detected the phosphorylation level of SHP2, an important downstream target of CD32b, and the results showed (Fig. 2G) that p-SHP2 level was highest in CD4^+^ T cells in the sFgl2-MSC coculture group, followed by the rFgl2 treatment group; and p-SHP2 level was lower in the groups with MSCs alone or CD32b blockade. Since SMAD2/3 is downstream of SHP2 [38–40], and its activation can promote the differentiation of Tregs [41, 42], we further detected the level of p-SMAD2/3. The results showed (Fig. 2H) that the p-SMAD2/3 level was the highest in sFgl2-MSCs, and decreased when CD32b was blocked or in the presence of MSCs or rFgl2 alone.

In conclusion, compared with MSCs or sFgl2 alone, sFgl2-MSCs have a more powerful immunosuppressive effect mediated by the promotion of SHP2 and SMAD2/3 phosphorylation, which can more effectively increase the proportion of Tregs and decrease the proportions of Th1 and Th17 cells.

### sFgl2-MSCs significantly alleviate experimental AIH in mice

**Fig. 3:**
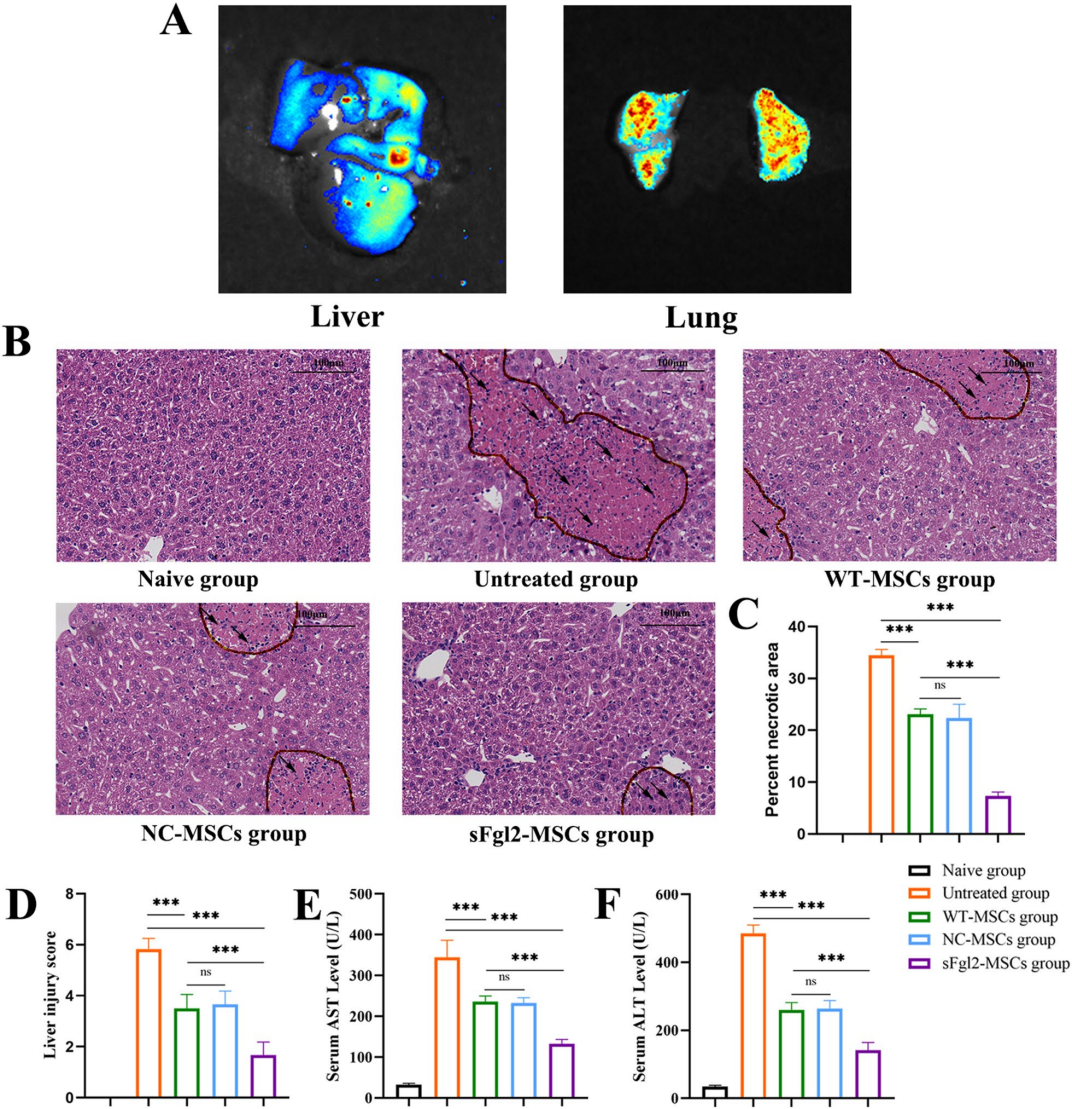
sFgl2-MSCs significantly alleviate liver injury in AIH mice. **A** Live organ imaging confirming that MSCs can reach the liver. **B** H&E staining showing pathological injuries in each group (20 ×). The black arrows show the positive staining areas. **C** Data display of liver necrosis area in each group, n = 6. **D** Liver damage was quantified in each group according to the Knodell scoring system, n = 6. **E** Serum AST levels in each group were determined by ELISA, n = 6. **F** Serum ALT levels in each group were determined by ELISA, n = 6. The differences between groups were calculated using ANOVA. ***: *P *< 0.001. Abbreviations: MSCs, mesenchymal stem cells; sFgl2, soluble fibronectin-like protein 2; WT-MSCs, wild-type MSCs; NC-MSCs, negative control MSCs; ALT, alanine transaminase; AST: aspartate transaminase

We found that sFgl2-MSCs had a more significant inhibitory effect than MSCs or sFgl2 alone, and the therapeutic effect of sFgl2-MSCs on AIH was verified in vivo. The concanavalin A (Con A)-induced mouse hepatitis model is an approved experimental AIH model [7]. Before the use of sFgl2-MSCs as an in vivo therapy, we first determined whether MSCs could reach the liver using living organ imaging technology. The results showed that although most of the MSCs were trapped in the lungs, some MSCs reached the liver (Fig. 3A). After 24 h, the liver was collected for histopathological analysis, and the results showed that the tissue damage in the untreated group was the most serious, as indicated by a large amount of inflammatory cell infiltration, large areas of liver necrosis and structural destruction. Compared with that in the untreated group, tissue damage in the MSC treatment group was reduced. In addition, inflammatory cells in the sFgl2-MSC group had the least infiltration and a more complete structure (Fig. 3B and C). In addition, the degree of liver damage was quantified by the Knodell score (Fig. 3D). Consistent with pathological findings, liver damage in mice treated with MSCs was significantly less severe than that in the untreated group (Fig. 3D, *P *< 0.001). Compared with that in the WT-MSC group, liver injury was further reduced in the sFgl2-MSC-treated group (Fig. 3D, *P *< 0.001).

AST and ALT can be used to evaluate liver function. Compared with those in the untreated group, the serum levels of AST (Fig. 3E, *P *< 0.001) and ALT (Fig. 3F, *P *< 0.001) in mice treated with MSCs were significantly decreased. The serum levels of AST (Fig. 3E, *P* < 0.001) and ALT (Fig. 3F, *P* < 0.001) were further decreased in mice treated with sFgl2-MSCs.

**Fig. 4:**
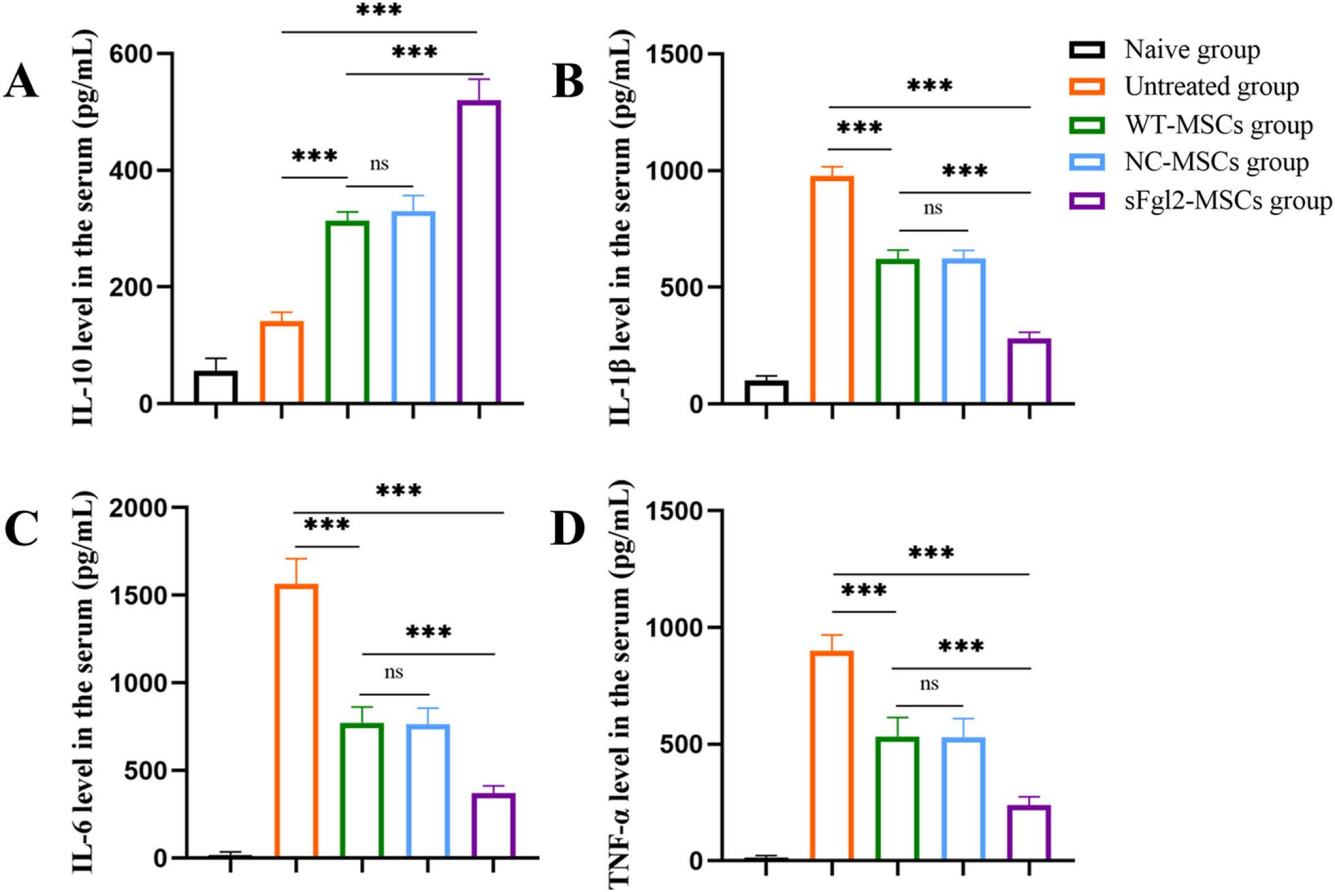
sFgl2-MSCs significantly regulate cytokines in AIH mice. **A**. ELISA was used to measure the expression level of IL-10 in the serum of each group. **B**. ELISA of the serum level of IL-1β in each group. ** C**. ELISA of the serum level of IL-6 in the various groups. **D**. ELISA of the serum levels of TNF-α in the various groups. The differences between groups were calculated using ANOVA. ***: *P *< 0.001. Abbreviations: MSCs, mesenchymal stem cells; sFgl2, soluble fibronectin-like protein 2; WT-MSCs, wild-type MSCs; NC-MSCs, negative control MSCs

Then, ELISA was used to assess inflammation in mice. Compared with that in the untreated group, the expression of anti-inflammatory cytokine IL-10 (Fig. 4A, *P* < 0.001) was significantly increased in the WT-MSC group, while the expression of the proinflammatory cytokines IL-1β (Fig. 4B, *P* < 0.001), IL-6 (Fig. 4C, *P* < 0.001) and TNF-α (Fig. 4D, *P*< 0.001) was decreased. Furthermore, the expression of IL-10 (Fig. 4A, *P *< 0.001) was further increased in the sFgl2-MSC treatment group compared with the WT-MSC treatment group, and the expression of IL-1β (Fig. 4B, *P*< 0.001), IL-6 (Fig. 4C, *P* < 0.001) and TNF-α (Fig. 4D, *P*< 0.001) was further decreased.

These experimental results show that sFgl2-MSCs have a more significant therapeutic effect on the treatment of experimental AIH than MSCs alone and can more effectively participate in the regulation of cytokines.

### sFgl2-MSCs decrease the proportion of CD4^+^ and CD8^+^ T cells in mice with experimental AIH

**Fig. 5:**
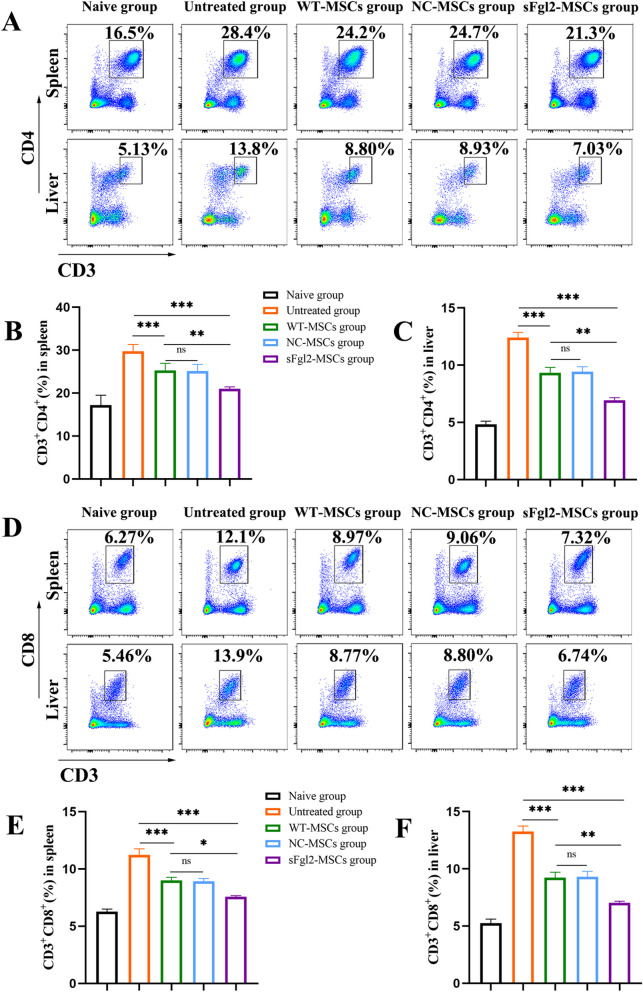
sFgl2-MSCs reduced the proportions of CD4^+^ T cells and CD8^+^ T cells in the spleen and liver in experimental AIH mice. **A** Dot plots showing CD4^+^ T cells in the spleen and liver. **B** The proportions of CD4^+^ T cells in the spleen in the different groups, n = 6. **C** The proportions of CD4^+^ T cells in the liver in the different groups, n = 6. **D** Dot plots showing CD8^+^ T cells in the spleen and liver. **E** The proportions of CD8^+^ T cells in the spleen in the different groups, n = 6. **F** The proportions of CD8^+^ T cells in the liver in the different groups, n = 6. *: *P *< 0.05, **: *P *< 0.01, ***: *P *< 0.001. The differences between groups were calculated using ANOVA. Abbreviations: MSCs, mesenchymal stem cells; sFgl2, soluble fibronectin-like protein 2; WT-MSCs, wild-type MSCs; NC-MSCs, negative control MSCs

CD4^+^ T cells and CD8^+^ T cells are the main T lymphocytes that infiltrate the liver during AIH [24] and mediate autoimmune hepatocyte injury. Therefore, the proportions of these cell types in the liver and spleen in experimental AIH mice were determined in vivo. The results showed that compared with those in the untreated group, the proportions of splenic CD4^+^ T cells (Fig. 5A and B, *P *< 0.001) and CD8^+^ T cells (Fig. 5D and E, *P *< 0.001) were significantly decreased by MSC treatment. After sFgl2-MSC treatment, the proportions of CD4^+^ T cells (Fig. 5A and B, *P *< 0.001) and CD8^+^ T cells (Fig. 5D and E, *P *< 0.001) were further decreased. To better examine the local therapeutic effect of sFgl2-MSCs on the liver, local lymphocytes in the liver were obtained for analysis. The results showed that compared with those in the untreated group, the proportions of liver-derived CD4^+^ T cells (Fig. 5A and C, *P *< 0.001) and CD8^+^ T cells (Fig. 5D and F, *P *< 0.001) were significantly decreased after treatment with MSCs. The proportions of CD4^+^ T cells (Fig. 5A and C, *P *< 0.001) and CD8^+^ T cells (Fig. 5D and F, *P *< 0.001) were further decreased in the sFgl2-MSC group.

These results suggest that sFgl2-MSCs could exert their effects throughout the body, reducing the proportions of CD4^+^ T cells and CD8^+^ T cells in the spleen and reducing the infiltration of CD4^+^ T cells and CD8^+^ T cells into the liver.

### sFgl2-MSCs increase the proportion of Tregs and decrease the proportion of Th17 cells

**Fig. 6:**
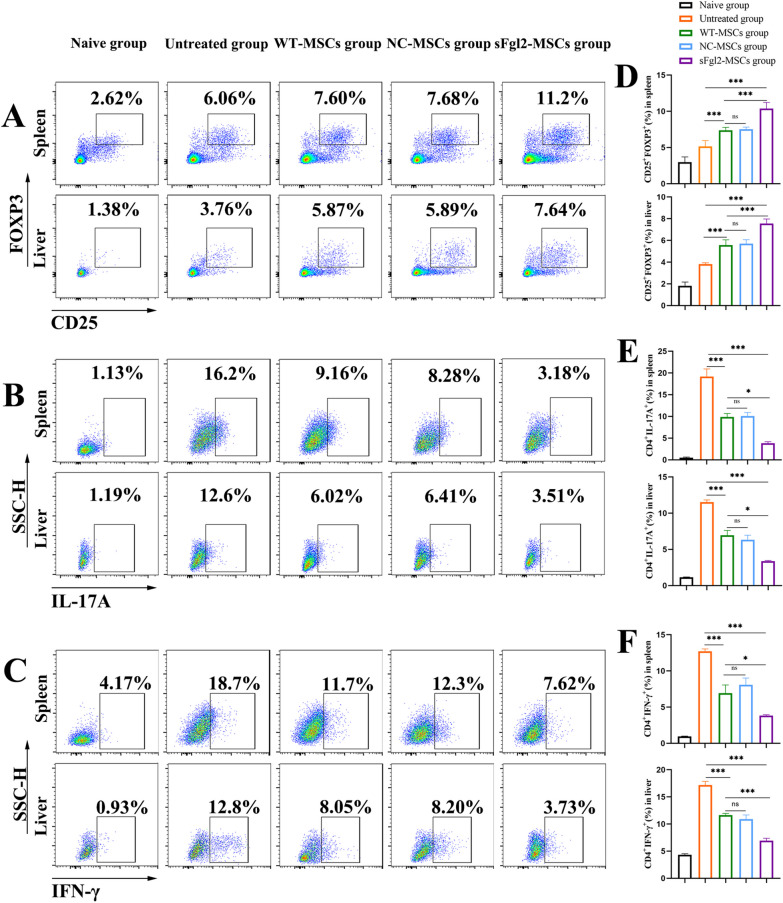
sFgl2-MSCs increased the proportions of Tregs in the spleen and liver in AIH mice and decreased the proportions of Th17 and Th1 cells. **A** Dot plots showing Tregs among CD4^+^ T cells in the spleen and liver. **B** Dot plots showing Th17 cells among CD4^+^ T cells in the spleen and liver. **C** Dot plots showing Th17 cells among CD4^+^ T cells in the spleen and liver. **D** The proportions of Tregs in the spleen and liver in the different groups, n = 6. **E** The proportions of Th17 cells in the spleen and liver in the different groups, n ≥ 5. **F** The proportions of Th1 cells in the spleen and liver in the different groups, n ≥ 5. The differences between groups were calculated using ANOVA. *: *P *< 0.05, **: *P *< 0.01, ***: *P *< 0.001. Abbreviations: MSCs, mesenchymal stem cells; sFgl2, soluble fibronectin-like protein 2; WT-MSCs, wild-type MSCs; NC-MSCs, negative control MSCs

Tregs, Th1 cells, and Th17 cells all play important roles in the development and treatment of AIH. Therefore, the proportions of these cell types in CD4^+^ T cells were determined in vivo. Compared with that in the untreated group, the proportion of Tregs (Fig. 6A and D, *P *< 0.001) in the spleen was increased after MSC treatment, while the proportions of Th17 cells (Fig. 6B and E, *P *< 0.001) and Th1 cells (Fig. 6C and F, *P *< 0.001) were decreased. Furthermore, the proportion of Tregs (Fig. 6A and D, *P *< 0.001) was further increased, while the proportions of Th17 cells (Fig. 6B and E, *P *< 0.001) and Th1 cells (Fig. 6C and F, *P *< 0.001) were further decreased after sFgl2-MSC treatment.

Then, the infiltration of Tregs, Th17 cells and Th1 cells in the liver was detected. The results showed that, compared with that in the untreated group, the proportion of Tregs (Fig. 6A and D, *P *< 0.001) in the liver was increased after MSC treatment, while the proportions of Th17 cells (Fig. 6B and E, *P *< 0.001) and Th1 cells (Fig. 6C and F, *P *< 0.001) were decreased. Furthermore, the proportion of Tregs (Fig. 6A and D, *P *< 0.001) was further increased, while the proportions of Th17 cells (Fig. 6B and E, *P *< 0.001) and Th1 cells (Fig. 6C and F, *P *< 0.001) were further decreased after sFgl2-MSC treatment.

**Fig. 7:**
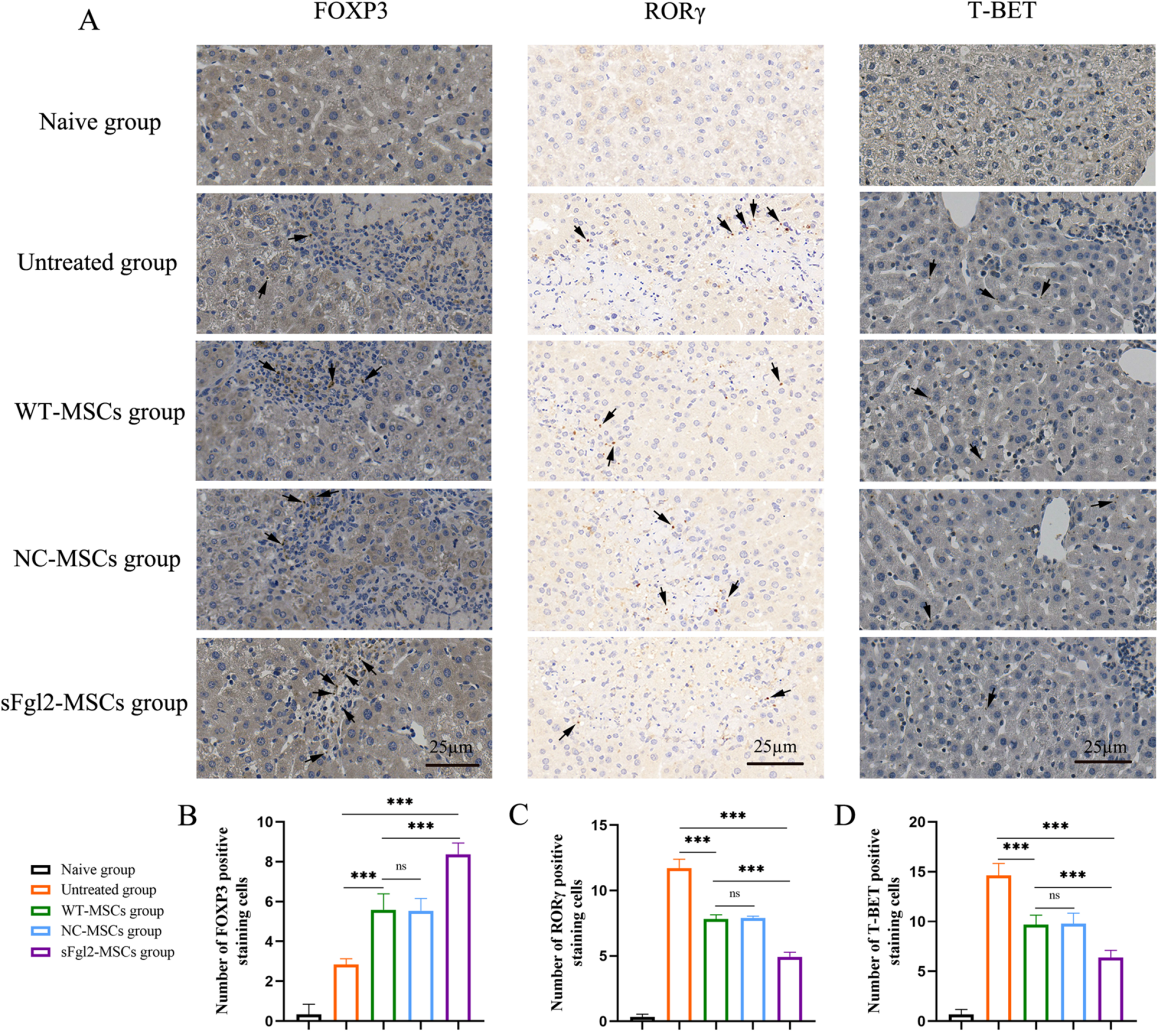
sFgl2-MSCs increased liver-infiltrating Foxp3-positive cells and reduced RORγ-positive cells and T-bet-positive cells. **A** Representative infiltration of Foxp3-, RORγ- and T-bet-positive cells in each group. **B** Data display of Foxp3-positive cells in each group, n = 6. **C** Data display of RORγ-positive cells in each group, n = 6. **D** Data display of T-bet-positive cells in each group, n = 6. The black arrows show the positive staining cells. The differences between groups were calculated using ANOVA. ***: *P *< 0.001. Abbreviations: MSCs, mesenchymal stem cells; sFgl2, soluble fibronectin-like protein 2; WT-MSCs, wild-type MSCs; NC-MSCs, negative control MSCs

To more intuitively observe the distribution of Tregs, Th1 cells and Th17 cells in the local liver, we used immunohistochemical staining to detect the specific markers of the above three T-cell subsets, among which T-bet is considered a Th1 cell marker [43, 44], Foxp3 is considered a marker of Tregs [45, 46], and RORγ is considered a marker of Th17 cells [46, 47]. The results showed (Fig. 7A) that compared with the untreated group, the number of Foxp3-positive cells in MSC-treated livers increased (Fig. 7B, *P *< 0.001), while the number of T-bet-positive cells and RORγ-positive cells decreased (Fig. 7C and D, *P *< 0.001). After sFgl2-MSC treatment, the number of Foxp3-positive cells further increased (Fig. 7B, *P *< 0.001), while the number of T-bet-positive cells and RORγ-positive cells further decreased (Fig. 7C and D, *P *< 0.001).

These results suggest that sFgl2-MSCs can increase the proportion of Tregs and decrease the proportions of Th17 cells and Th1 cells to reduce the systemic and local immune response to injury.

## Discussion

Glucocorticoids alone or in combination with azathioprine are the standard treatment for AIH and are effective in most patients [6]. The treatment end point of AIH is the normalization of serum transaminase and immunoglobulin levels and is used to define complete response [48, 49]. Additional treatments need to be developed for patients who do not have remission or cannot tolerate standard treatments, and stem cell therapy is a potential important treatment. MSCs can regulate the differentiation and activation of almost all immune cells, such as T cells, macrophages and DCs, and are easy to culture, expand and preserve in vitro. Due to their excellent immunomodulatory effects, MSCs have been widely used in the treatment of T-cell-mediated hepatitis [9, 20, 50–52]. However, the curative effect of MSCs alone is unsatisfactory, so it is necessary to find ways to enhance the immune regulatory function of MSCs [18–20].

FcγRIIb, which is the only inhibitory Fcγ receptor [21], is thought to be present on B cells, macrophages, DCs, and granulocytes. FcγRIIb is associated with immune tolerance and can promote the apoptosis of B cells [53]. In addition, the absence of CD32b increases the risk of autoimmune diseases [54, 55]. Recent studies have shown that this receptor is also present on T cells [22, 56, 57]. In this study, the expression of CD32b on CD4^+^ T cells increased after murine MSCs were cocultured with CD4^+^ T cells in vitro. Unlike other Fcγ receptors, the functional ligand of CD32b is sFgl2 [22]. sFgl2 has a wide range of effects on cells: it promotes apoptosis in B cells, inhibits the maturation of DCs, promotes the polarization of alternatively activated macrophages, and is an important molecule that mediates the effect of Tregs [58, 59]. sFgl2 can inhibit the proliferation and differentiation of T lymphocytes, and Fgl2 plays a key role in AIH by regulating Tregs and inhibiting Th17 cells and Th1 cells [60].

Based on the plasticity of MSCs, modifying MSCs to overexpress related factors is a common strategy to enhance the immunomodulatory function of MSCs [61, 62]. Therefore, in this study, the Fgl2 gene was transfected into MSCs by lentiviral transfection technology to induce high sFgl2 overexpression, and the expression of CD32b was induced to the same extent after MSCs were cocultured with CD4^+^ T cells. Furthermore, we also found that the proportion of Tregs among CD4^+^ T cells increased after the cells were cocultured with transfected or untransfected MSCs, and the increase was most significant in the sFgl2-MSC treatment group, while the decrease in Th1 and Th17 cells was the most significant. This result suggests that MSCs or sFgl2 alone can exert immunosuppressive effects to different degrees, thereby increasing the proportion of Tregs and decreasing the proportions of Th17 and Th1 cells. In addition, sFgl2-MSCs have a more significant immunoregulatory effect than unmodified MSCs.

Next, we proposed a possible mechanism by which sFgl2-MSCs can more effectively regulate CD4^+^ T-cell differentiation by regulating the levels of p-SHP2 and p-SMAD2/3. CD32b contains the immunoreceptor tyrosine-based inhibitory motif domain, which is an inhibitory domain [63], and can activate the activation of downstream SHP2 [53]. In fact, Hou et al. [64] reported that sFgl2 activates the ERK1/2-STAT3 pathway by activating SHP2, thereby biasing macrophages toward a pro-repair phenotype, which is associated with endometriosis progression, and our study also revealed that the level of p-SHP2 increased in the presence of sFgl2, and the level of p-SHP2 was the highest in the sFgl2-MSC treatment group because MSCs induced the expression of CD32b in CD4^+^ T cells; the level of p-SHP2 decreased after blocking CD32b. SMAD2/3 is closely related to the differentiation of Tregs [41, 42] and is also a reported important downstream target of SHP2. This study revealed that MSCs or rFgl2 can increase the level of p-SMAD2/3, which was further increased after sFgl2-MSC treatment. Therefore, sFgl2-MSCs exhibit more powerful immune regulatory function by effectively activating SHP2 and SMAD2/3.

Previous studies have confirmed that MSCs can accumulate in large numbers in the lungs, and some MSCs can migrate to other sites, such as the liver [65–67], and MSCs will preferentially gather at the injury site [68, 69]. Our live organ imaging results suggest that although many MSCs accumulate in the lungs, some MSCs reach the liver as the site of injury and play a role.

The efficacy of MSCs accumulated in the lungs is also worthy of attention. Zhang X et al. found that prostaglandin E2 produced by lung MSCs promoted the synthesis and release of acetylcholine, activated cholinergic anti-inflammatory pathways, reduced lung inflammation, and relieved respiratory symptoms in patients with acute respiratory distress syndrome [70]. Furthermore, S.H.M. Pang et al. It was found that apoptosis and exocytosis of lung MSCs can cause changes in alveolar macrophage metabolism and inflammatory pathways, thereby affecting immunosuppression and reducing disease severity [71]. In addition, MSCs have a paracrine function and can secrete a variety of molecules including prostaglandin E2 to play a role [72]. The sFgl2-MSCs transformed in this study can also secrete sFgl2, and after accumulating in the lungs, they can also secrete sFgl2 to play an immunosuppressive function.

In fact, the results of this study showed that compared with the MSCs-treated group, sFgl2-MSCs more significantly protected the liver structure, significantly reduced inflammatory cell infiltration, lowered serum AST and ALT levels, and effectively reduced the pro-inflammatory factor IL-6 compared with the MSCs-treated group., IL-1β and TNF-α levels, while increasing the level of the anti-inflammatory factor IL-10, reducing systemic inflammation, these results suggest that sFgl2-MSCs have a more potent therapeutic effect on experimental AIH.

CD4^+^ T cells and CD8^+^ T cells are the main T lymphocytes that infiltrate the liver [1]. The in vivo results confirmed that in experimental AIH mice, CD4^+^ T cells and CD8^+^ T cells were significantly elevated in the spleen and liver, indicating systemic inflammation. After sFgl2-MSC treatment in experimental AIH mice, CD4^+^ T cells and CD8^+^ T cells were significantly decreased compared with those in the MSC group.

Tregs, which make up 5–10% of all peripheral T cells in healthy humans, control innate and acquired immune responses by limiting tolerance mechanisms and thereby limiting the ability of circulating autoreactive T cells to damage tissue [6]. However, in AIH, the number and functionality of Tregs are decreased [73, 74]. Adoptive transfer of Tregs has shown initial success in treating experimental AIH [75]. Therefore, increasing the number and function of Tregs is critical for treatment [6]. We found that the proportion of Tregs was significantly increased in the spleen and liver after treatment with sFgl2-MSCs compared with MSCs. In addition, increases in the proportions of Th1 cells and Th17 cells, as well as the levels of corresponding cytokines interferon-γ and IL-17A, are characteristic of AIH [4, 76]. The loss of Th17 cells has been shown to reduce T-cell-mediated hepatitis [77]. In this study, we found that compared with MSC treatment, sFgl2-MSC treatment further reduced the proportions of Th17 cells and Th1 cells in the spleen and liver. Therefore, it is reasonable to conclude that sFgl2-MSCs play a significant role in the treatment of AIH by regulating the differentiation of Tregs, Th17 cells and Th1 cells in vivo.

Although this study showed that MSCs increased CD32b expression on CD4^+^ T cells, the specific mechanism still needs to be further examined. To further improve the therapeutic effect of sFgl2-MSCs, we will further optimize their application strategy in follow-up studies to enhance the curative effect of sFgl2-MSCs on AIH.

## Conclusion

**Fig. 8:**
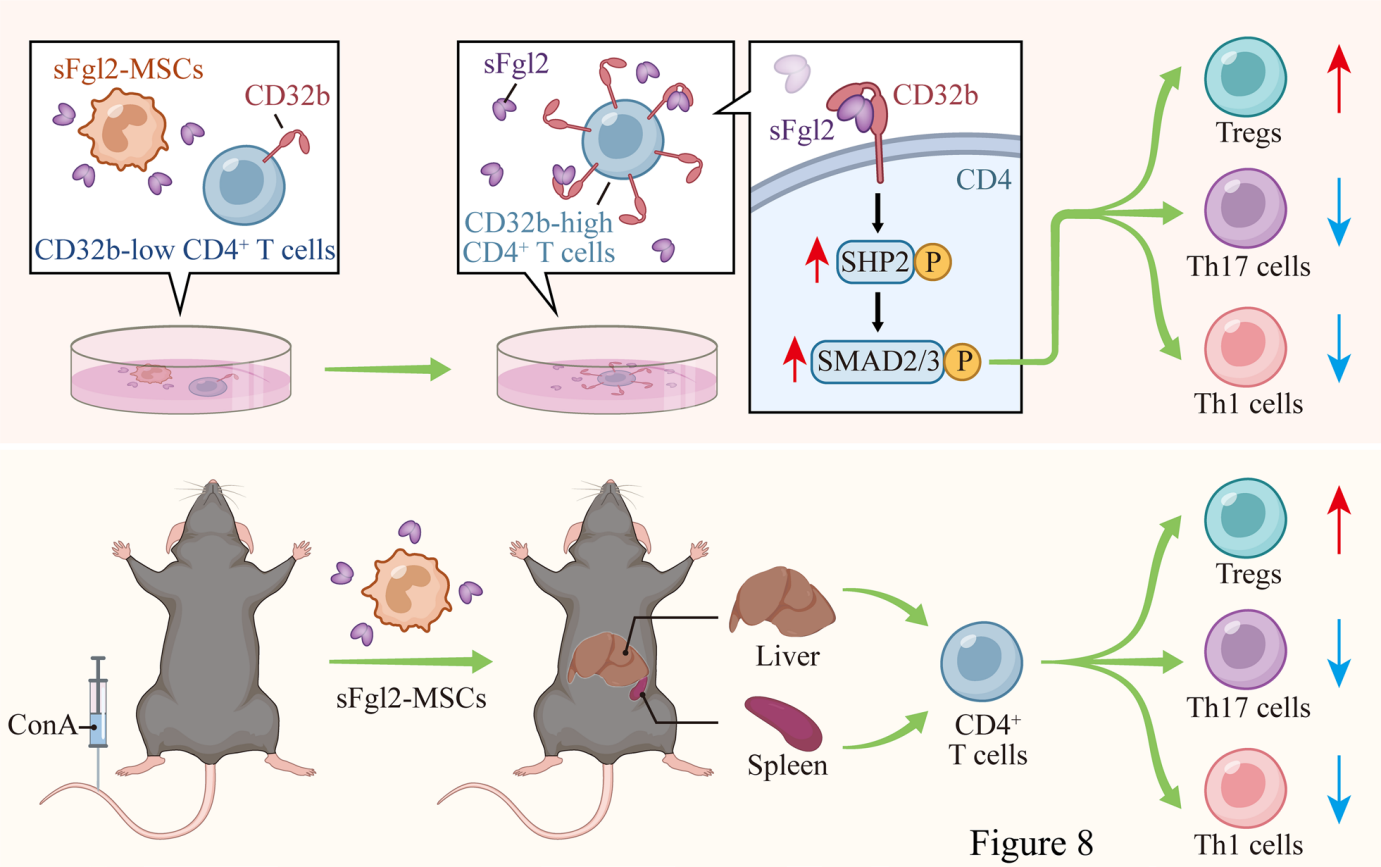
Possible mechanism of sFgl2 gene-modified MSCs in the treatment of experimental AIH mice. sFgl2 gene-modified MSCs may promote the differentiation of CD4^+^ T cells into Tregs and inhibit their differentiation into Th17 and Th1 cells by activating SHP2 and SMAD2/3 to treat experimental AIH mice more effectively. AIH: autoimmune hepatitis; MSCs: mesenchymal stem cells; sFgl2: soluble fibronectin-like protein 2; sFgl2-MSCs: sFgl2 gene-modified MSCs; Tregs: T regulatory cells. This image was drawn by the author using Adobe Illustrator 2023

sFgl2 gene-modified MSCs may promote the differentiation of Treg cells by enhancing the phosphorylation of SHP2 and SMAD2/3, inhibit the differentiation of Th17 and Th1 cells, and have a significant immune regulation effect (Fig. 8), so as to better treat experimental AIH mice, and it provides a possible strategy for the clinical treatment of AIH.

### Supplementary Information

**Additional file 1: Fig. S1.** The original blots presented in Fig. 2G.

**Additional file 2: Fig. S2.** The original blots presented in Fig. 2H.

## Data Availability

All data generated or analyzed during this study are included in this article.

## Abbreviations

AIH: Autoimmune hepatitis
MSCs: Mesenchymal stem cells
sFgl2: Soluble fibronectin-like protein 2
rFgl2: Recombinant Fgl2
WT-MSCs: Wild-type MSCs
NC-MSCs: Negative control MSCs
Con A: Concanavalin A
ELISA: Enzyme-linked immunosorbent assay
FBS: Fetal bovine serum
MOI: Multiplicity of infection
IFN-γ: Interferon-γ
IL: Interleukin
ANOVA: Analysis of variance
TNF-α: Tumor necrosis factor-α
Tregs: T regulatory cells
ALT: Alanine transaminase
AST: Aspartate transaminase
GFP: Green fluorescent protein
PE: Phycoerythrin
APC: Allophycocyanin
FITC: Fluorescein isothiocyanate
PBS: Phosphate-buffered saline

## References

[1] Senaldi G, Portmann B, Mowat AP, Mieli-Vergani G, Vergani D (1992). Immunohistochemical features of the portal tract mononuclear cell infiltrate in chronic aggressive hepatitis. Archiv Dis Childhood.

[2] Schümann J, Wolf D, Pahl A, Brune K, Papadopoulos T, van Rooijen N, et al. Importance of Kupffer Cells for T-Cell-Dependent Liver Injury in Mice. *The American journal of pathology* (2000) 157(5):1671–83. . doi: 10.1016/s0002-9440(10)64804-3. 10.1016/S0002-9440(10)64804-3 PMC1885735 11073826

[3] Liberal R, Grant CR, Mieli-Vergani G, Vergani D (2013). Autoimmune hepatitis: a comprehensive review. J Autoimmun.

[4] Longhi MS, Ma Y, Mieli-Vergani G, Vergani D (2010). Aetiopathogenesis of autoimmune hepatitis. J Autoimmun.

[5] Liberal R, Krawitt EL, Vierling JM, Manns MP, Mieli-Vergani G, Vergani D (2016). Cutting edge issues in autoimmune hepatitis. J Autoimmun.

[6] Mieli-Vergani G, Vergani D, Czaja AJ, Manns MP, Krawitt EL, Vierling JM (2018). Autoimmune hepatitis. Nat Rev Dis Primers.

[7] Manns MP, Lohse AW, Vergani D (2015). Autoimmune hepatitis-update 2015. J Hepatol.

[8] Ichai P, Samuel D (2008). Etiology and prognosis of fulminant hepatitis in adults. Liver Transp Off Publ Am Assoc Study Liver Dis Int Liver Transpl Soc.

[9] Tamura R, Uemoto S, Tabata Y (2016). Immunosuppressive effect of mesenchymal stem cell-derived exosomes on a concanavalin a-induced liver injury model. Inflam Regener.

[10] Kanazawa H, Fujimoto Y, Teratani T, Iwasaki J, Kasahara N, Negishi K (2011). Bone marrow-derived mesenchymal stem cells ameliorate hepatic ischemia reperfusion injury in a rat model. PLoS ONE.

[11] Bartholomew A, Sturgeon C, Siatskas M, Ferrer K, McIntosh K, Patil S (2002). Mesenchymal stem cells suppress lymphocyte proliferation in vitro and prolong skin graft survival in vivo. Exp Hematol.

[12] Halim NSS, Ch'ng ES, Kardia E, Ali SA, Radzi R, Yahaya BH (2019). Aerosolised mesenchymal stem cells expressing angiopoietin-1 enhances airway repair. Stem Cell Rev Rep.

[13] Qi K, Li N, Zhang Z, Melino G (2018). Tissue regeneration: the crosstalk between mesenchymal stem cells and immune response. Cell Immunol.

[14] Shi Y, Su J, Roberts AI, Shou P, Rabson AB, Ren G (2012). How mesenchymal stem cells interact with tissue immune responses. Trends Immunol.

[15] Alfaifi M, Eom YW, Newsome PN, Baik SK (2018). Mesenchymal stromal cell therapy for liver diseases. J Hepatol.

[16] Lee KC, Lin HC, Huang YH, Hung SC (2015). Allo-transplantation of mesenchymal stem cells attenuates hepatic injury through il1ra dependent macrophage switch in a mouse model of liver disease. J Hepatol.

[17] Kubo N, Narumi S, Kijima H, Mizukami H, Yagihashi S, Hakamada K (2012). Efficacy of adipose tissue-derived mesenchymal stem cells for fulminant hepatitis in mice induced by concanavalin A. J Gastroenterol Hepatol.

[18] Nauta AJ, Westerhuis G, Kruisselbrink AB, Lurvink EG, Willemze R, Fibbe WE (2006). Donor-derived mesenchymal stem cells are immunogenic in an allogeneic host and stimulate donor graft rejection in a nonmyeloablative setting. Blood.

[19] Plumas J, Chaperot L, Richard MJ, Molens JP, Bensa JC, Favrot MC (2005). Mesenchymal stem cells induce apoptosis of activated T cells. Leukemia.

[20] Pan L, Liu C, Liu Q, Li Y, Du C, Kang X (2021). Human Wharton's jelly-derived mesenchymal stem cells alleviate concanavalin a-induced fulminant hepatitis by repressing Nf-Κb signaling and glycolysis. Stem Cell Res Ther.

[21] Nimmerjahn F, Ravetch JV (2008). Fcgamma receptors as regulators of immune responses. Nature Rev Immunol.

[22] Morris AB, Farley CR, Pinelli DF, Adams LE, Cragg MS, Boss JM (2020). Signaling through the Inhibitory Fc Receptor Fcγriib Induces Cd8(+) t cell apoptosis to limit T Cell immunity. Immunity.

[23] Cherny I, Shepshelovich D, Lahav M, Rabizadeh E (2019). the effect of infectious and autoimmune diseases on procoagulant activity of fibrinogen-like protein 2 in the peripheral blood. Harefuah.

[24] Liu XG, Liu Y, Chen F (2017). Soluble fibrinogen like protein 2 (Sfgl2), the novel effector molecule for immunoregulation. Oncotarget.

[25] Hu J, Yan J, Rao G, Latha K, Overwijk WW, Heimberger AB (2016). The duality of Fgl2 - secreted immune checkpoint regulator versus membrane-associated procoagulant: therapeutic potential and implications. Int Rev Immunol.

[26] Fu Y, Ding Y, Wang Q, Zhu F, Tan Y, Lu X (2020). Blood-stage malaria parasites manipulate host innate immune responses through the induction of Sfgl2. Sci Adv.

[27] Lee HJ, Jung H, Kim DK (2021). Ido and Cd40 may be key molecules for immunomodulatory capacity of the primed tonsil-derived mesenchymal stem cells. Int J Mol Sci.

[28] Zhao N, Li H, Yan Y, Jiang R, He X (2017). Mesenchymal stem cells overexpressing Il-35 effectively inhibit Cd4(+) T cell function. Cell Immunol.

[29] Zhao W, Wang Y, Wang D, Sun B, Wang G, Wang J (2008). Tgf-Beta expression by allogeneic bone marrow stromal cells ameliorates diabetes in nod mice through modulating the distribution of Cd4+ T cell subsets. Cell Immunol.

[30] Gong B, Zheng L, Lu Z, Huang J, Pu J, Pan S (2021). Mesenchymal stem cells negatively regulate Cd4(+) T Cell activation in patients with primary Sj&Ouml;Gren syndrome through the Mirna-125b and Mirna-155 Tcr pathway. Mol Med Rep.

[31] Tiegs G, Hentschel J, Wendel A (1992). A T cell-dependent experimental liver injury in mice inducible by concanavalin A. J Clin Invest.

[32] Wang H, Feng X, Yan W, Tian D (2020). Regulatory T cells in autoimmune hepatitis: unveiling their roles in mouse models and patients. Front Immunol.

[33] Bozza M, Bliss JL, Maylor R, Erickson J, Donnelly L, Bouchard P (1999). Interleukin-11 reduces T-cell-dependent experimental liver injury in mice. Hepatology.

[34] Han X, Yang Q, Lin L, Xu C, Zheng C, Chen X (2014). Interleukin-17 enhances immunosuppression by mesenchymal stem cells. Cell Death Differ.

[35] Shan Z, Liu X, Chen Y, Wang M, Gao YR, Xu L (2018). Chitinase 3-like-1 promotes intrahepatic activation of coagulation through induction of tissue factor in mice. Hepatology.

[36] Ye K, Lan X, Wang G, Zhang B, Xu X, Li X (2018). B7–H1 expression is required for human endometrial regenerative cells in the prevention of transplant vasculopathy in mice. Stem Cells Int.

[37] Wang H, Zhao Y, Ren B, Qin Y, Li G, Kong D (2021). Endometrial regenerative cells with galectin-9 high-expression attenuate experimental autoimmune hepatitis. Stem Cell Res Ther.

[38] Liu YN, Guan Y, Shen J, Jia YL, Zhou JC, Sun Y (2020). Shp2 positively regulates cigarette smoke-induced epithelial mesenchymal transition by mediating Mmp-9 production. Respir Res.

[39] Xu J, Tao B, Guo X, Zhou S, Li Y, Zhang Y (2017). Macrophage-restricted Shp2 tyrosine phosphatase acts as a rheostat for Mmp12 through Tgf-Β activation in the prevention of age-related emphysema in mice. J Immunol.

[40] Lu YG, Tan H, Ma Q, Li XX, Cui J, Zhang X (2021). Sh2 domain-containing protein tyrosine phosphatase-2 (Shp-2) prevents cardiac remodeling after myocardial infarction through Erk/Smad signaling pathway. Hum Cell.

[41] An T, Guo M, Fan C, Huang S, Liu H, Liu K (2023). Sfgl2-Treg positive feedback pathway protects against atherosclerosis. Int J Mol Sci.

[42] Iizuka-Koga M, Nakatsukasa H, Ito M, Akanuma T, Lu Q, Yoshimura A (2017). Induction and maintenance of regulatory T Cells by transcription factors and epigenetic modifications. J Autoimmun.

[43] Qian Y, Pei Y, Jiang W, Zheng C (2022). Astilbin improves pregnancy outcome in rats with recurrent spontaneous abortion by regulating Th1/Th2 balance. Immunopharmacol Immunotoxicol.

[44] Zhang W, Pan Y, Gou P, Zhou C, Ma L, Liu Q (2018). Effect of xanthohumol on Th1/Th2 balance in a breast cancer mouse model. Oncol Rep.

[45] Farag AGA, Maraee AH, Rifaat Al-Sharaky D, Elshaib ME, Kohla MSM, Shehata WA (2021). Tissue expression of Il-17a and Foxp3 in acne vulgaris patients. J Cosmet Dermatol.

[46] Wang C, Huang CF, Li M (2022). Sodium houttuynia alleviates airway inflammation in asthmatic mice by regulating Foxp3/rorgammat expression and reversing Treg/Th17 cell imbalance. Int Immunopharmacol.

[47] Menter T, Hayoz S, Zucca E, Kimby E, Dirnhofer S, Tzankov A (2020). Immunomodulatory drugs may overcome the negative prognostic role of active Th17 axis in follicular lymphoma: evidence from the Sakk35/10 trial. Br J Haematol.

[48] Manns MP, Czaja AJ, Gorham JD, Krawitt EL, Mieli-Vergani G, Vergani D (2010). Diagnosis and management of autoimmune hepatitis. Hepatology.

[49] Easl Clinical Practice Guidelines (2015). Autoimmune hepatitis. J Hepatol.

[50] Masalova OV, Lesnova EI, Klimova RR, Ivanov AV, Kushch AA (2021). Mesenchymal stem cells can both enhance and inhibit the cellular response to DNA immunization by genes of nonstructural proteins of the hepatitis C Virus. Int J Mol Sci.

[51] Shen S, Dai H, Fei Z, Chai Y, Hao Y, Fan Q (2021). Immunosuppressive nanoparticles for management of immune-related adverse events in liver. ACS Nano.

[52] Zhao J, Li Y, Jia R, Wang J, Shi M, Wang Y (2021). Mesenchymal stem cells-derived exosomes as dexamethasone delivery vehicles for autoimmune hepatitis therapy. Front Bioeng Biotechnol.

[53] Pearse RN, Kawabe T, Bolland S, Guinamard R, Kurosaki T, Ravetch JV (1999). Ship recruitment attenuates Fc gamma Riib-induced B cell apoptosis. Immunity.

[54] Floto RA, Clatworthy MR, Heilbronn KR, Rosner DR, MacAry PA, Rankin A (2005). Loss of function of a lupus-associated fcgammariib polymorphism through exclusion from lipid rafts. Nat Med.

[55] Kono H, Kyogoku C, Suzuki T, Tsuchiya N, Honda H, Yamamoto K (2005). Fcgammariib Ile232thr transmembrane polymorphism associated with human systemic lupus erythematosus decreases affinity to lipid rafts and attenuates inhibitory effects on B cell receptor signaling. Human Mol Genet.

[56] Campos Carrascosa L, van Beek AA, de Ruiter V, Doukas M, Wei J, Fisher TS (2020). Fcγriib engagement drives agonistic activity of fc-engineered Αox40 antibody to stimulate human tumor-infiltrating T cells. J Immunother Cancer.

[57] Holgado MP, Sananez I, Raiden S, Geffner JR, Arruvito L (2018). Cd32 ligation promotes the activation of Cd4(+) T Cells. Front Immunol.

[58] Shalev I, Liu H, Koscik C, Bartczak A, Javadi M, Wong KM (2008). Targeted deletion of Fgl2 leads to impaired regulatory T cell activity and development of autoimmune glomerulonephritis. J Immunol.

[59] Shah FH, Kim SJ (2021). Targeting Fgl2, a molecular drug target for glioblastoma, with natural compounds through virtual screening method. Fut Med Chem.

[60] Ai G, Yan W, Yu H, Xiao F, Xi D, Ma K (2018). Soluble Fgl2 restricts autoimmune hepatitis progression via suppressing Tc17 and conventional Cd8+ T Cell function. J Gene Med.

[61] Mun JY, Shin KK, Kwon O, Lim YT, Oh DB (2016). Minicircle microporation-based non-viral gene delivery improved the targeting of mesenchymal stem cells to an injury site. Biomaterials.

[62] Liao W, Pham V, Liu L, Riazifar M, Pone EJ, Zhang SX (2016). Mesenchymal stem cells engineered to express selectin ligands and Il-10 exert enhanced therapeutic efficacy in murine experimental autoimmune encephalomyelitis. Biomaterials.

[63] Ono M, Bolland S, Tempst P, Ravetch JV (1996). Role of the inositol phosphatase ship in negative regulation of the immune system by the receptor Fc(Gamma)Riib. Nature.

[64] Hou XX, Wang XQ, Zhou WJ, Li DJ (2021). Regulatory T cells induce polarization of pro-repair macrophages by secreting Sfgl2 into the endometriotic milieu. Commun Biol.

[65] Chen D, Li Q, Meng Z, Guo L, Tang Y, Liu Z (2017). Bright polymer dots tracking stem cell engraftment and migration to injured mouse liver. Theranostics.

[66] Gholamrezanezhad A, Mirpour S, Bagheri M, Mohamadnejad M, Alimoghaddam K, Abdolahzadeh L (2011). In vivo tracking of 111in-oxine labeled mesenchymal stem cells following infusion in patients with advanced cirrhosis. Nucl Med Biol.

[67] Devine SM, Cobbs C, Jennings M, Bartholomew A, Hoffman R (2003). Mesenchymal stem cells distribute to a wide range of tissues following systemic infusion into nonhuman primates. Blood.

[68] Li Y, Chen J, Chen XG, Wang L, Gautam SC, Xu YX (2002). Human marrow stromal cell therapy for stroke in rat: neurotrophins and functional recovery. Neurology.

[69] Yin Y, Hao H, Cheng Y, Gao J, Liu J, Xie Z (2018). The homing of human umbilical cord-derived mesenchymal stem cells and the subsequent modulation of macrophage polarization in type 2 diabetic mice. Int Immunopharmacol.

[70] Zhang X, Wei X, Deng Y, Yuan X, Shi J, Huang W (2022). Mesenchymal stromal cells alleviate acute respiratory distress syndrome through the cholinergic anti-inflammatory pathway. Signal Trans Target Ther.

[71] Pang SHM, D'Rozario J, Mendonca S, Bhuvan T, Payne NL, Zheng D (2021). Mesenchymal stromal cell apoptosis is required for their therapeutic function. Nature Commun.

[72] Liu J, Gao J, Liang Z, Gao C, Niu Q, Wu F (2022). Mesenchymal stem cells and their microenvironment. Stem Cell Res Ther.

[73] Longhi MS, Ma Y, Bogdanos DP, Cheeseman P, Mieli-Vergani G, Vergani D (2004). Impairment of Cd4(+)Cd25(+) regulatory T-cells in autoimmune liver disease. J Hepatol.

[74] Longhi MS, Ma Y, Mieli-Vergani G, Vergani D (2012). Regulatory T cells in autoimmune hepatitis. J Hepatol.

[75] Yoshizawa K, Joshita S, Matsumoto A, Umemura T, Tanaka E, Morita S (2016). Incidence and prevalence of autoimmune hepatitis in the Ueda Area, Japan. Hepatol Res Off J Japan Soc Hepatol.

[76] Behfarjam F, Nasseri-Moghaddam S, Jadali Z (2019). Enhanced Th17 responses in patients with autoimmune hepatitis. Middle East J Digest Dis.

[77] Lafdil F, Wang H, Park O, Zhang W, Moritoki Y, Yin S (2009). Myeloid Stat3 inhibits T cell-mediated hepatitis by regulating T helper 1 cytokine and interleukin-17 production. Gastroenterology.

