# Supplementary figures and images for: sFgl2 gene-modified MSCs regulate the differentiation of CD4+ T cells in the treatment of autoimmune hepatitis
Source: Stem Cell Res Ther. 2023 Nov 3;14:316. doi: 10.1186/s13287-023-03550-x (PMC10625288; doi:10.1186/s13287-023-03550-x)

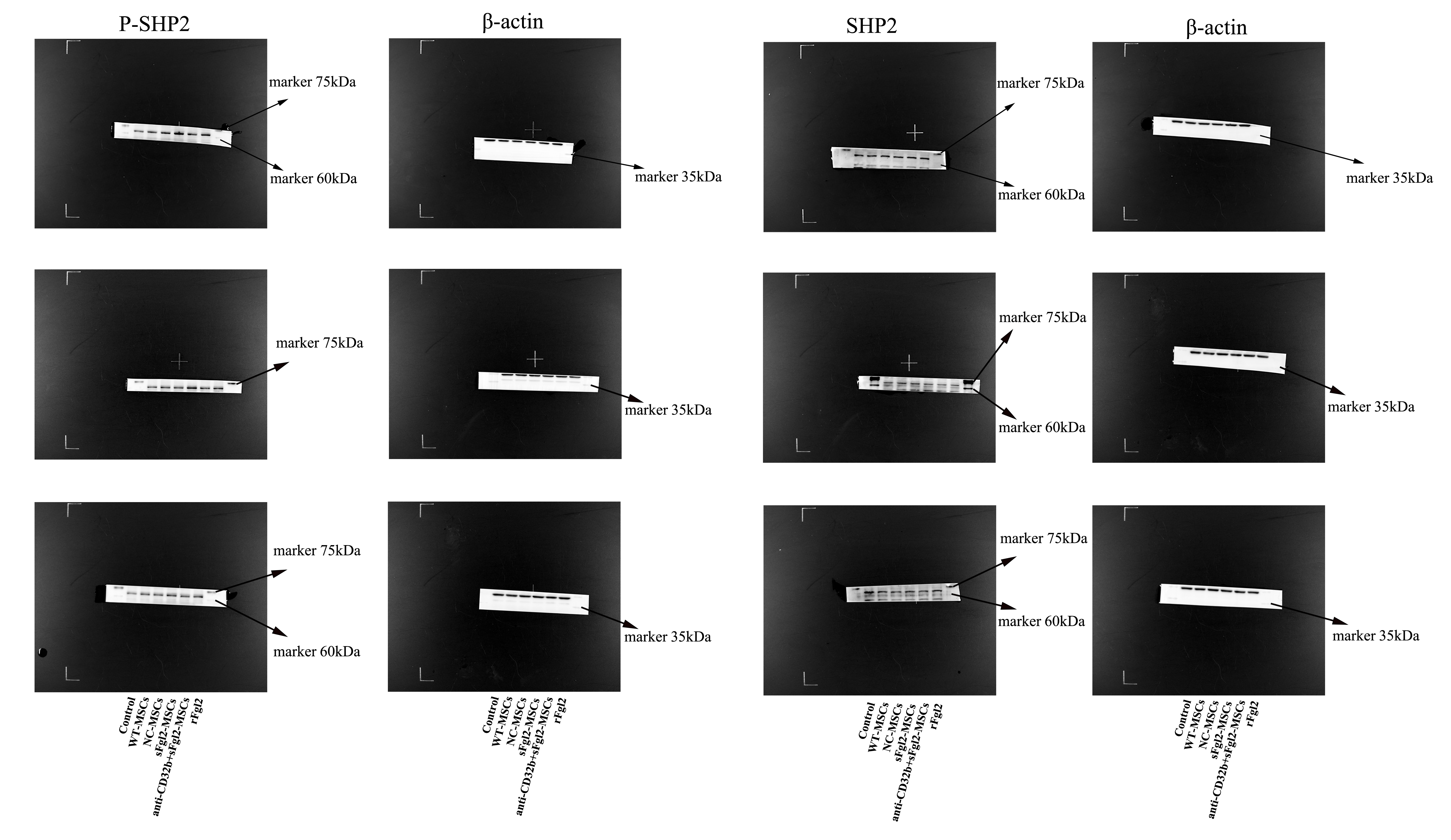

Supplement: Supplementary file 1 — Additional file 1: Fig. S1. The original blots presented in Fig. 2G. [file 13287_2023_3550_MOESM1_ESM.tif]

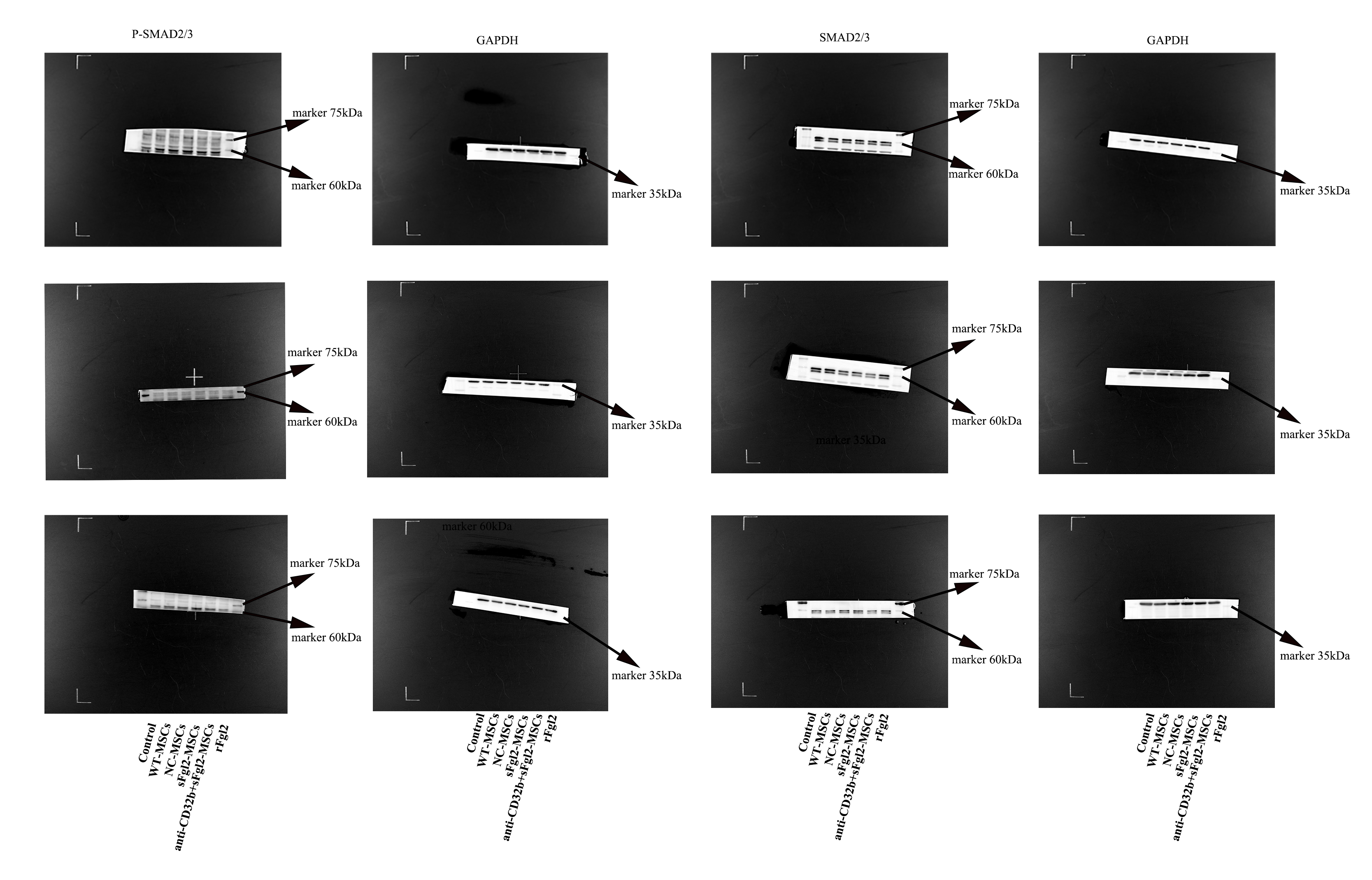

Supplement: Supplementary file 2 — Additional file 2: Fig. S2. The original blots presented in Fig. 2H. [file 13287_2023_3550_MOESM2_ESM.tif]
